# The Impact of Risk Perception on Social Distancing during the COVID-19 Pandemic in China

**DOI:** 10.3390/ijerph17176256

**Published:** 2020-08-27

**Authors:** Kefan Xie, Benbu Liang, Maxim A. Dulebenets, Yanlan Mei

**Affiliations:** 1 School of Management, Wuhan University of Technology, Wuhan 430070, China; xkf@whut.edu.cn (K.X.); liangbenbu@whut.edu.cn (B.L.); 2 Institute of Wuhan Studies, Jianghan University, Wuhan 430056, China; 3 Department of Civil & Environmental Engineering, Florida A&M University-Florida State University (FAMU-FSU) College of Engineering, Tallahassee, FL 32310-6046, USA; 4 School of Resource and Environmental Engineering, Wuhan University of Science and Technology, Wuhan 430081, China; meiyanlan@wust.edu.cn; 5 Evergrande School of Management, Wuhan University of Science and Technology, Wuhan 430065, China

**Keywords:** social distancing, risk perception, safety climate, COVID-19 pandemic, public health

## Abstract

Social distancing is one of the most recommended policies worldwide to reduce diffusion risk during the COVID-19 pandemic. Based on a risk management perspective, this study explores the mechanism of the risk perception effect on social distancing in order to improve individual physical distancing behavior. The data for this study were collected from 317 Chinese residents in May 2020 using an internet-based survey. A structural equation model (SEM) and hierarchical linear regression (HLR) analyses were conducted to examine all the considered research hypotheses. The results show that risk perception significantly affects perceived understanding and social distancing behaviors in a positive way. Perceived understanding has a significant positive correlation with social distancing behaviors and plays a mediating role in the relationship between risk perception and social distancing behaviors. Furthermore, safety climate positively predicts social distancing behaviors but lessens the positive correlation between risk perception and social distancing. Hence, these findings suggest effective management guidelines for successful implementation of the social distancing policies during the COVID-19 pandemic by emphasizing the critical role of risk perception, perceived understanding, and safety climate.

## 1. Introduction

As the number of global coronavirus cases explodes rapidly, threatening millions of lives, the COVID-19 pandemic has become the fastest spreading, most extensive, and most challenging public health emergency worldwide since World War II [1]. Compared to seasonal influenza, this coronavirus appears to be more contagious and transmits much faster. For example, the basic reproduction rate R_0_ for seasonal influenza is approximately 1.28, while for COVID-19, this value comprises 3.3 on average [2,3,4]. With no efficacious treatments and vaccines available yet, social distancing measures are still one of the common approaches to reduce the rate of infection. Moreover, for the foreseeable multiple waves of the pandemic, COVID-19 prevention will continue to rely on physical distancing behaviors until safe vaccines or effective pharmacological interventions become accessible. Accordingly, social distancing has been implemented by authorities across the globe to prevent diffusion of the disease.

Facing this global pandemic, even each government has issued advice about mobility restriction, the definition of social distancing, and distancing rules. However, the guidance documents differ around the world. For example, China, Japan, and other Asian countries opted for lockdowns and strict liberty-limiting during coronavirus outbreaks. In contrast, several countries, such as the United States (U.S.) and Brazil, suffer more from this pandemic due to relaxed social distancing. The possible reason for this is that people have different risk perceptions and viewpoints towards the pandemic because of differences in cultural backgrounds across these countries [5]. Additionally, different countries proposed different physical distancing rules, like keeping at least a 2 m (6 feet) distance (in the U.S. or the United Kingdom (U.K.)), a 1.5 m distance (in Australia), or a 1 m distance (in China and South Africa) from others (see Table 1). One of the potential concerns might arise due to the differences in perception of various individuals regarding the potential distance over which the coronavirus can transmit. Other reasons such as religion, culture, customs, and even population and economy, could determine social distancing rules as well [6]. Multiple studies have generated ample evidence to support the 1~2 m social distancing rule from observations and simulations [7]. Despite the existing differences in awareness of the pandemic, social distancing practices should be effectively implemented to stop the ongoing spread of COVID-19 [8].

**Figure 1:**
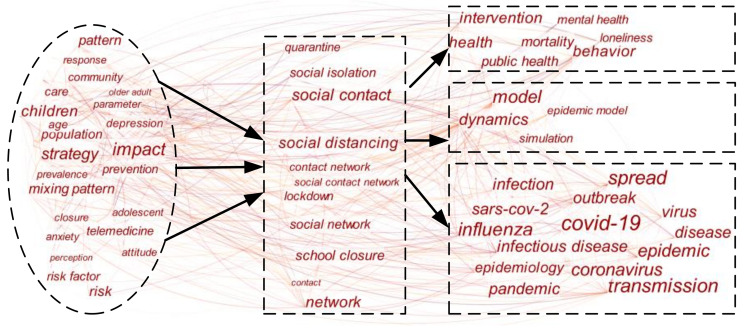
Keywords co-occurrence networks of social distancing research.

Social distancing has received increasing attention in numerous studies over recent decades, especially since the COVID-19 outbreaks. In order to explore critical points and network patterns of these prior research studies, a co-word analysis was conducted. The literature keywords present the relationship between the study subjects and a concentration of the research content [18]. Hence, the application of a co-word analysis on the existing literature can provide generic knowledge and network patterns in the studies on social distancing. An integrated search was conducted given the topic of social distancing, such as “physical distancing”, “social isolation”, “lockdown”, etc. Subsequently, 978 related papers published from 1 January 2000 through 28 June 2020 were retrieved using the Web of Science core database. Then, using Citespace software, which is designed as a tool for progressive knowledge domain visualization [19], the co-occurrence matrix of keywords was calculated and visualized, as shown in Figure 1. The size of the keywords presents the frequency of co-occurrence and the connection shows the significance of co-occurrence [20].

Based on the co-word analysis, the major research focus and inner bibliometric characteristics of social distancing were concluded from four perspectives, such as how social distancing affects the pandemic, the additional effects and challenges caused by social distancing, modeling and simulation of social distancing, and influencing factors. Most of the previous studies [21] confirmed that social distancing has positive effects on the pandemic slowdown while several studies [22] seem not to confirm this. Some studies believe that social distancing cuts off the transmission path of the virus, thereby reducing R_0_ [23]. Moreover, different mathematical models and simulations have displayed a good correlation with the data showed in biomedical studies, which offered a high level of evidence for the impact of social distancing measures to contain the pandemic [24,25]. For example, based on simple stochastic simulations, Cano et al. [26] evaluated the efficiency of social distancing measures to tackle the COVID-19 pandemic. Okuonghae and Omame [27] found if at least 55% of the population would implement social distancing measures, the pandemic will eventually disappear according to the numerical simulations of the model. Nevertheless, a systematic review and meta-analysis demonstrated that the social distancing regulation showed a non-significant protective effect, which can be caused by the persisting knowledge gaps in disparate population groups [22].

Although various cohort studies and modeling simulations have found that the social distance regulations can effectively prevent the spread of the pandemic, the additional effects and challenges caused by social distancing cannot be ignored. For instance, anxiety associated with social distancing may have a long-term effect on mental health [28] and social inequality. Furthermore, loneliness pandemics are arising from physical isolation as well [29]. As a form of reduced movement and face-to-face connections between people, social distancing has changed residents’ conventional health behaviors, which may lead to increasing obesity, accidental pregnancies, and other health risks [30,31]. A national survey carried out in Italy demonstrated that individual needs shifted towards the three bottom levels of the Maslow’s pyramid (i.e., belongingness and love needs, safety needs, and physiological needs) due to the social isolation [32].

Compared with the impact of social distancing, more previous studies focused on its influencing factors. First, at the national and cultural dimension levels, Akim and Ayivodji [33] concluded that certain economic and fiscal interventions were associated with higher compliance with social distancing. Huynh [5] found that countries with higher “Uncertainty Avoidance Index” indicate a lower proportion of public gatherings. Likewise and Moon [34] explored the role of cultural orientations and showed that vertical collectivism predicted stronger compliance with social distancing norms. Then, at the level of public society, Aldarhami et al. [35] conducted a survey indicating that the high level of public awareness affects social distancing implementation. Besides, public health authorities and experts alike pointed out that mass media and information played an important role in developing public awareness and constructing social distancing behaviors among social populations [36]. Lastly, from the perspective of individual behaviors and psychological factors, OConnell et al. [1] reported that more antisocial individuals may pose a health risk to the public and engage in fewer social distancing regulations. Based on a cross-sectional online survey, Yanti et al. [37] identified that the respondents who had sufficient knowledge and a good attitude would positively comply with safety behaviors, such as keeping a physical distance from others and wearing face masks in public places.

Although the evidence unambiguously supported that implementing the social distancing regulations has a crucial effect on restraining the pandemic [38], recent studies found that mobility restrictions do not lead to an expected reduction of coronavirus cases [8,39]. Previous literature has conducted various analyses regarding the different factors motivating social distancing behaviors. However, facing the current enormous gap between the method and the existing practice, limited research has paid attention to the key factors from the perspective of risk management. Because of the significant role that individuals and public awareness play in compliance with social distancing, this study focuses on the mechanism of the risk perception effect on social distancing. Individual’s perceived understanding and safety climate are also examined to identify their effectiveness in the relationship between risk perception and social distancing. Based on a quantitative online survey with a sample size of 317 participants from China over the period of May 2020, we built the structural equation model (SEM) and conducted hierarchical linear regression (HLR) analysis to examine how the selected moderators influence social distancing behavior.

The remainder of the paper is organized as follows. Section 2 will review the risk perception theories and develop several hypotheses with the conceptual framework. Section 3 describes the research methodology, data collection, and measurement of latent variables. Then, we analyze the data and examine hypotheses (Section 4) and finally, discuss the implications and limitations of our findings (Section 5) as well as draw the main conclusions (Section 6).

## 2. Hypothesis Development and Study Model

### 2.1. Risk Perception and Perceived Understanding Related to Social Distancing

Risk exists objectively, but distinct people will take different behavioral decisions when they perceive risk differently [40]. Hence, even many medical experts stressed the importance of maintaining physical distancing amid the COVID-19 pandemic and people’s risk perception still colors beliefs about facts. The concept of risk perception differs among different disciplines [41]. In this study, risk perception in the context of the pandemic is defined as the psychological processes of subjective assessment of the probability of being infected by the coronavirus, an individual’s perceived health risk, and available protective measures [42,43]. Compared to the concept of risk perception in other fields, the health risk perception and the severity caused by the consequences of subsequent behavioral decisions are the most prominent features. Empirical evidence has indicated that health risk perception may significantly affect people’s self-protective behaviors and increase negative consequences of health risks [44]. Dionne et al. [45] found that risk perception associated with medical activities was a critical predictor of the epidemic prevention behaviors. Accordingly, as reported, underestimation of the pandemic knowledge and health risks could lead to decreasing implementation of social distancing.

Most previous research focused on identifying influencing factors for people’s health risk perception as risk perception largely determines whether individuals would take protective measures during the pandemic. Also, there are various factors that reduce the substantial deviation between the actual objective risk and subjective feelings. Perceived understanding is just one of the crucial factors that refers to situational awareness for the adoption of healthcare protections when facing the pandemic [46]. According to the theory of planned behavior, only when people realize that they are in a health risk or even death risk will they have the situational awareness to take further healthcare protections. Effective and timely perceived understanding will greatly promote people to translate risk perception into actual actions [47]. Perceived understanding plays a vital role in the adoption of healthcare behaviors. Therefore, the following four hypotheses were developed, considering the findings from previous studies.

**Hypothesis** **1 (H1).** *The level of health Risk Perceptions about the COVID-19 pandemic has a positive effect on the adoption of Social Distancing behavior.*

**Hypothesis** **2 (H2).** *The level of health Risk Perception has a positive effect on Perceived Understanding during the COVID-19 pandemic.*

**Hypothesis** **3 (H3).** *The level of Perceived Understanding about the COVID-19 pandemic has a positive effect on the adoption of Social Distancing behavior.*

**Hypothesis** **4 (H4).** *Perceived Understanding about the COVID-19 pandemic plays a mediating role between Risk Perception and Social Distancing behavior.*

### 2.2. Risk Perception and Safety Climate Related to Social Distancing

Facing huge economic pressure and public opinion, many companies and organizations gradually re-opened. At the same time, these institutions require their employees to implement the social distancing policies strictly. Similarly, when people go out to eat, shop, and entertain, many public places remind people to maintain a physical distance. Regardless of whether it is a social organization or a public place, this kind of a reminder message released through information media has virtually created a safe climate to require people to take necessary measures and reduce the spread of the virus. Generally, the safety climate refers to individuals’ perception of safety regulations, procedures, and behaviors in the workplace [48]. From the perspective of pandemic prevention and control, the safety climate relates to a consensus created by the work environment which will promote people consciously or unconsciously to take the appropriate safety measures. Namely, safety climate reflects common awareness among employees on the importance of organizational safety issues [49].

**Figure 2:**
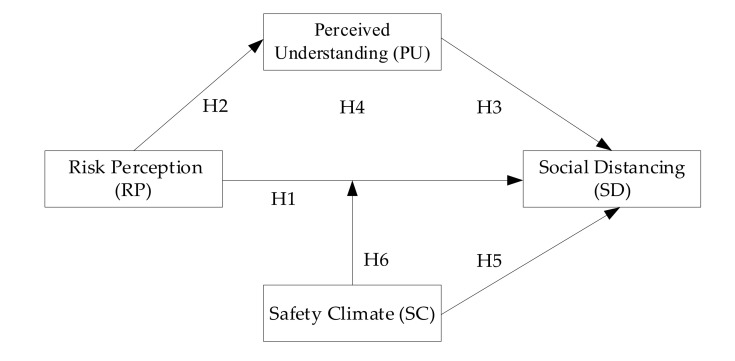
Conceptual framework.

Numerous observations and studies attest to the relationship between safety climate and protective behavior. Bosak et al. [50] found that a good safety climate was negatively related to people’s risk behaviors. Moreover, another study showed that safety climate completely mediated the effect of risk perception on safety management [49]. However, few studies focused on the influence of safety climate on people’s self-protection behavior during the pandemic. Taking protective measures, such as social distancing, wearing face masks, and other self-prevention behaviors, are instrumental to avoid the spread of the infection. An organization with a good safety climate can carry out relevant safety training and drills, so as to suppress the potential risk tendency and promote their employees’ safety behaviors. Therefore, if the working environment can strengthen the education and publicity of pandemic knowledge, people are more willing to take correct protective measures, such as maintaining a social distance. Additionally, Koetke et al. [51] also pointed out that safety climate (trust in science) played a moderating role in the relationship between conservative and social distancing intentions. To conclude, based on the above literature reviews, the conceptual framework of this study is illustrated in Figure 2. Our last two hypotheses read as follows:

**Hypothesis** **5 (H5).** The level of Safety Climate about the COVID-19 pandemic has a positive effect on the adoption of Social Distancing behavior.

**Hypothesis** **6 (H6).** Safety Climate positively moderates the impact of Risk Perception on Social Distancing behavior during the COVID-19 pandemic.

## 3. Materials and Methods

### 3.1. Population and Sample

According to the 44th China Statistical Report on Internet Development, which was announced by the China Internet Network Information Center (CNNIC), in 2019, there were 854 million internet users in China. Several studies exploring some physical or psychological influencing mechanisms, such as risk perception, showed no significant difference between internet users and non-users [52]. Therefore, online questionnaires were randomly collected from internet users through wenjuan.com. A total of 317 completed responses were received with an effective rate of 94.63%, after excluding suspected unreal answers completed in less than 60 s. Additionally, participants were first directed to review and provide their consent using an online informed consent form, which was pre-approved by a panel of experts and the institutional review board, before answering the survey questionnaire. The data collection was anonymously conducted throughout May 2020.

The female participants constituted 48.3% of the sample, while 51.7% of the sample were male participants. Among the respondents, most of them were young people, 31.9% belonged to the age group of 18–24 years, while 40.7% belonged to the age group of 25–39 years. A total of 84.5% of the participants had a college degree or above and only 6% had a lower level education than high school. Out of the total sample, 48.6% reported to be living in rural areas and 51.4% lived in urban communities. It should be noticed that there were 15.14% of the participants living in Hubei Province, which used to be the epicenter of the COVID-19 pandemic in China.

### 3.2. Survey Instrument

The initial questionnaire contained 22 questions to measure these 4 latent variables, including Risk Perception—RP (7 items), Perceived Understanding—PU (4 items), Social Distancing—SD (5 items), and Safety Climate—SC (6 items). All the measurement items were prepared based on the review of related literature and methods (Table 2). For example, initial items for RP were generated following previous questionnaires conducted by Dionne et al. [45] and Kim et al. [53]. Measurement items of PU were compiled based on the infectious disease-specific health literacy scale [54] and the study by Qazi et al. [46]. The SC instrument statements were taken from the literature review and previously completed research [51,55,56]. Based on the studies of Swami et al. [57] and Gudi et al. [58], initial measurement questions of SD were developed. Additionally, to ensure the validity of the draft questionnaire, the original survey instrument statements were revised based on the suggestions from a panel of experts, including 5 professionals of risk management, 5 public health specialists, and 5 community managers. Then, necessary modifications were made by simplifying, rewording, and replacing several items after 15 experts reviewed the survey structure, wording, and item allocation. According to the expert panel’s feedback, the item-level content validity index (I-CVI) of the 18 items were all greater than 0.78 and the scale-level CVI (S-CVI) is 0.97 (>0.90), indicating an excellent validity of this scale (see Supplementary Materials). An initial survey with 22 items was first pilot tested among a randomly selected sample of 100 internet users. After conducting cognitive interviews with the pilot sample participants and analyzing the reliability and correlations, 4 measurement items (RP5, RP6, RP7, and SD5) with a item-to-total correlation below 0.5 were removed. Finally, a formal questionnaire containing 18 items was developed.

The response scale for all the survey items was a 5-point Likert scale with categories ranging from 1 = “strongly disagree” to 5 = “strongly agree”. All of the items were phrased positively, so that a higher score represented stronger agreement. Table 2 displays an overview of the scale and questionnaire items.

### 3.3. Data Analysis

Descriptive statistics and correlation analyses of the latent variables were first examined. Then, the exploratory factor analysis (EFA) and the confirmatory factor analysis (CFA) were conducted to verify the unidimensionality and reliability of the measurement items. The SEM can be applied to control for measurement errors as well as to use parameters to identify interdependencies [2,50]. Hence, this approach is appropriate to test the hypotheses by conducting the path analyses. In addition, to examine the moderating effect, HLR was carried out to verify Hypotheses H5 and H6. Amos version 24.0 software was applied for CFA and SEM (Hypotheses H1–H4). The remaining analyses, e.g., EFA and HLR (Hypotheses H5 and H6), were done using SPSS 22.0. (IBM, Armonk, NY, USA)

## 4. Results

The means, standard deviations (S.D.), and inter-correlations of all the measures are contained in Table 3. There are significant positive correlations between the four variables. RP has significant positive correlations with SD and PU, suggesting a partial support for Hypotheses H1 and H2, respectively. Moreover, both PU and SC showed a significant positive correlation with SD, indicating that Hypotheses H3 and H5 were partially supported as well.

### 4.1. Reliability and Validity Analysis

Reliability can be formally defined as the proportion of observed score variance, which is attributable to the true score variance. There exist several approaches to evaluate the reliability of a measuring item and internal consistency is the most widely used method in research with a cross-sectional design. The Cronbach’s alpha (α) can be used to estimate the internal consistency [59]. A standard value for Cronbach’s alpha is 0.70 or above, which indicates strong internal consistency of adopted scales [60]. Table 4 indicates that all four latent variables have good reliability (Cronbach’s α > 0.7), suggesting that the measurement items are appropriate indicators of their respective constructs.

The validity analysis is used to examine the accuracy of the measurement instrument, namely the validity of the scale. The validity analysis mainly includes the content validity and the construct validity, of which the content validity has been supported by the expert panel’s recommendations and pre-tests, while the construct validity requires a combination of EFA and CFA. First, the Kaiser-Meyer-Olkin (KMO) test value was 0.888. In addition, the result of the Bartlett test (χ^2^ = 3135.94, *df* = 153, *p* < 0.001) was large and significant. Hence, the data shown in Table 4 were suitable for CFA. Then, the measurement items identified four factors that exactly correspond to four latent variables. These four factors explained 66.41% of the total variance. Similarly, the CFA results confirmed the four-factor model. In this study, the goodness-of-fit statistics were found to be X^2/^*df* = 2.776 (<3.0), GFI = 0.889 (>0.80), CFI = 0.928 (>0.90), IFI = 0.929 (>0.90), TLI = 0.911 (>0.90), AGFI = 0.847 (>0.80), PGFI = 0.644 (>0.5), and RMSEA = 0.075 (<0.08). Summing up the factor analysis results, the survey items are appropriate to conceptualize the latent variables. Based on the results of CFA, the congeneric reliability (CR) and the average variance extracted (AVE) can be calculated by Formulas (1) and (2):

(1) $$CR=\frac{{\left(\sum_{i=1}^{k}\lambda_{i}\right)}^{2}}{{\left(\sum_{i=1}^{k}\lambda_{i}\right)}^{2}+\sum_{i=1}^{k}\sigma_{e_{i}}^{2}}$$

(2) $$AVE=\frac{\sum_{i=1}^{k}\lambda^{2}{}_{i}}{k},$$

where $\lambda_{i}$ and $\sigma_{e_{i}}^{2}$ represent the regression weight (factor loading) and measure variance estimate of the measurement item *i*, respectively, and *k* is the number of measurement items. *CR* and *AVE* are other effective measures to evaluate the construct validity. Correspondingly, according to Jobson [61], the acceptable value of *CR* is 0.7 and above, while *AVE* should be 0.5 and above. Table 4 demonstrates that most of the values of *CR* and *AVE* met the standards, suggesting an acceptable goodness-of-fit for the further SEM analysis.

### 4.2. SEM Analysis

**Figure 3:**
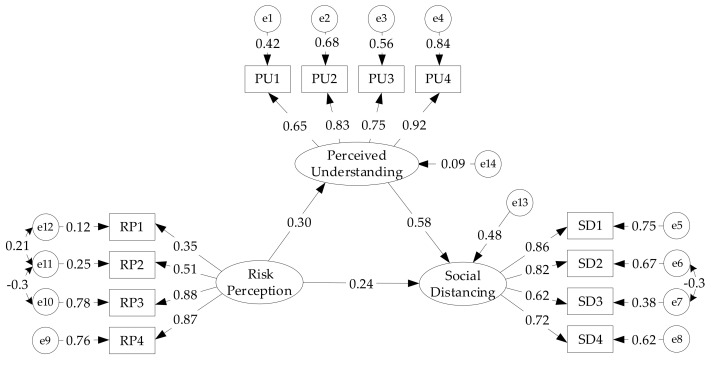
Standard loads, errors, and standardized regression weights of the structural equation model (SEM).

Based on the conceptual framework, the SEM analysis was conducted to explore the relationship between RP, SD, and PU (as the mediator). The hypothesized model shown in Figure 3 was first examined. Table 5 summarizes the fit indices of the model, which indicates an excellent goodness-of-fit for the data based on the majority of indices. In this model, several path analyses were developed to test Hypotheses H1, H2, and H3. As shown in Table 6, RP has significant positive relationships with PU (β = 0.296, C.R. = 4.435, *p* < 0.001) and SD (β = 0.238, C.R. = 4.421, *p* < 0.001). Likewise, PU plays a significant positive role on SD (β = 0.581, C.R. = 8.426, *p* < 0.001) as well. Thus, it implies that Hypotheses H1, H2, and H3 are supported.

Bias-corrected (BC) and percentile (PC) bootstrapping approaches were carried out to verify the mediating effect of PU. Previous studies have found that bootstrapping was a proper method that can provide a robust test of mediating hypotheses [62]. Accordingly, the significant effect of risk perception on social distancing could be assessed through perceived understanding by using the bootstrapping of 5000 sub-samples. As can be seen from Table 7, the values of the lower and upper limits (95% BC and PC bootstrap confidence intervals) for the indirect effect (β = 0.100) were all greater than zero. Moreover, the value of Z (indirect effect/standard error) equals 2.5 (>1.96). Subsequently, similar to an indirect effect, it was found that there were no zero values between the lower and upper limits (95% BC and PC bootstrap confidence intervals) for the direct effect (β = 0.138, Z = 3.45). Therefore, perceived understanding partially mediates the positive effects of risk perception on social distancing. In other words, perceived understanding did not completely offset the effect of risk perception, which partially explains the social distancing. In summary, these results confirmed Hypothesis H4.

### 4.3. HLR Analysis

Hypothesis H6 predicted that safety climate positively moderates the impact of risk perception on social distancing. To test the moderation effects, the HLR analysis was conducted. Model 1 serves as a baseline with independent variables RP and SC. Then, model 2 incorporated additional variables RP×SC. Table 8 presents the significant interaction effects of the two-way interaction effect between RP and SC on SD (Model 2, RP×SC, β = −0.242, *p* < 0.001). As shown in Table 8, while risk perception is positively associated with social distancing regardless of the value of safety climate, the safety climate further reduces the positive effect. Thus, Hypothesis H6 is partially supported. Additionally, whether SC is in Model 1 (β = 0.566, *p* < 0.001) or Model 2 (β = 0.4689, *p* < 0.001), it presents a statistically significant positive relationship with SD, which further supports Hypothesis H5.

## 5. Discussion

This study has continued to demonstrate that social distancing behaviors play a critical role in preventing the diffusion of the COVID-19 pandemic. In identifying influencing factors that lead to social distancing, previous studies have highlighted risk perception as a leading indicator of protective behaviors [42,44,45]. People should be encouraged to promote risk perception in order to identify and rectify infection risks and health issues related to unprotected behaviors during the COVID-19 pandemic. However, limited research has examined whether different risk perception of individuals affects their interpretation of the social distancing regulations in an equivalent manner. By investigating the measurement scales of risk perception, perceived understanding, safety climate, and social distancing across populations of internet users in China, this study addressed the mechanism of the risk perception effect on social distancing to improve individuals’ physical distancing behaviors.

### 5.1. Research and Policy Implications

This study provided evidence that risk perception and perceived understanding can significantly affect people’s social distancing behaviors during the COVID-19 pandemic. The results of the path analysis supported Hypotheses H1, H2, and H3. It is evident from Figure 3, Table 5, and Table 6 that the path coefficients are significant and the overall hypothesized model has a good fit for the investigation. These findings are in line with Aldarhami et al. [35], Zhong et al. [63], and Machida et al. [64]. A key principle of social distancing behavior is that risk perception is a critical condition for protective action. The results support the finding that higher risk perception motivates people to comply with social distancing. Only by enhancing risk perception can people truly remain vigilant against the pandemic and take protective measures. Therefore, when the government implements social distancing and other prevention measures, it must take into account the public risk perception and improve public environmental awareness through various means, such as social media, press conferences, standard therapy, and guidelines for the outbreak response. In particular, it is necessary to rectify pandemic rumors to prevent incorrect information that can potentially reduce public risk perception.

Besides, we confirmed a dual effect of perceived understanding on social distancing. First, perceived understanding was found to predict social distancing directly. These results are consistent with other studies [1,46] which have shown that increased perceived understanding can encourage people to gain more knowledge about the pandemic and health risks, so that they would engage more in the social distancing regulations. Then, we identified that perceived understanding as a factor showed an incomplete mediating effect on the relationship between risk perception and social distancing. Previous literature regarding perceived understanding shows that it affects the social distancing behaviors related to the sources of information [46]. On the other hand, our results confirm an indirect positive effect of risk perception on social distancing through perceived understanding. Hence, with the help of the authority of medical experts, we should promptly popularize scientific knowledge of the pandemic and prevention measures among communities to enhance public perceived understanding. In addition, the increase in risk perception can promote public desire to understand the pandemic and pay more attention to their own health risks. The authorities should improve pandemic information release channels.

Moreover, we identified that a positive perception of safety climate (*β* = 0.566, *p* < 0.001) would promote adherence to social distancing and that this effect would be stronger than the risk perception (*β* = 0.165, *p* < 0.001). This finding concurs with the study conducted by Kouabenan et al. [49]. The achievement of a consensus on a safe climate requires the joint efforts of the organization and society. First, workplaces such as shops, cafeterias, office spaces, and public transit systems have to strengthen pandemic prevention and control drills. Then, it is necessary to support community propaganda and scientific knowledge popularization and gather the individual consensus on self-protective behaviors. It is also strongly recommended to wear a face mask, keep a 2 m physical distance between workers, and use sanitary measures in public venues.

**Figure 4:**
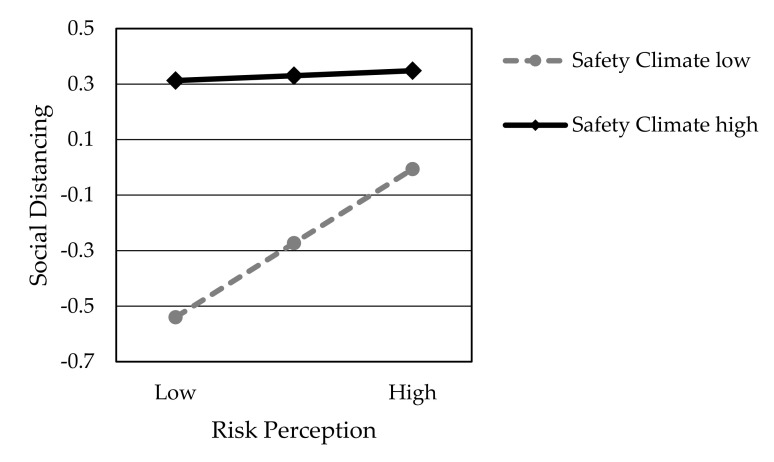
Safety Climate as a function of the interaction between Risk Perception and Social Distancing.

Finally, we demonstrated that safety climate, risk perception, and social distancing are the interacting factors, supporting our hypothesis that a moderating effect of safety climate on the relationship between risk perception and social distancing exists, as found in Kouabenan et al. [49], Bosak et al. [50], and Koetke et al. [51] (see Hypothesis H6). However, we did not find that safety climate increased the degree to which the risk perception positively affects social distancing. As shown in Figure 4, risk perception was positively related to social distancing under the conditions of a high safety climate as well as under the conditions of a low safety climate. More importantly, we found that safety climate is a factor that lessens the positive correlation between risk perception and social distancing. This moderating effect improves our understanding of the contexts in which risk perception affects social distancing. Yet, as described by Kouabenan et al. [49], the safety climate was viewed as the key factor because it completely mediated the effect of perceived risk on safety behavior.

One potential explanation for this difference of findings is the complex content of safety climate measurement items, because it actually includes three clauses. Compared to the previous studies, we regarded the safety climate as the whole of social consciousness. The overall promotion of social protection awareness will replace the role of risk perception and may lead to compliance with social distancing through the public herd effect. Therefore, while focusing on the importance of risk perception, we cannot ignore the positive incentives for social distancing brought by a good safety climate. In addition to enhancing employees’ consensus on pandemic prevention, qualified organizations can physically isolate workspaces and public venues in time and space. For example, people should avoid going out for mass gatherings (lunches, shopping, traveling, education, leisure, etc.). Then, for management commitment, they should physically divide the restaurant space, office space, and other public areas to ensure that people have sufficient isolation distance. Flexible work scheduling, online office hours, and e-learning are encouraged for implementation. Conclusively, application of innovating social distance management technologies (e.g., technologies that are based on an emerging range of ICT technologies [65] like Bluetooth, Radio Frequency Identification, Cloud Mobile, and others) can assist with achieving an accurate measurement of the physical distance between individuals and momentarily reminding people to maintain a social distance as needed. In public venues, such as dining areas, using multimedia, posters, and ground stickers with social distancing reminders can create a good safety climate.

### 5.2. Limitations and Future Research

Although substantial efforts were put into this study to ensure the reliability and validity of the results, a few limitations still exist, which might be explored in further research. First, our sample does consist of Chinese internet users but may not have all the attributes that perfectly match the characteristics of the current Chinese population. Without collecting data from other regions and having a representative sample, the generalizability of our findings is limited to a certain extent. A cross-regional, more representative study with a bigger sample size could be used in future studies in order to improve accuracy and generalizability of the results. Second, we measured all the latent variables with a simple one-dimensional factor by using a cross-sectional design. The results could neither exclude the possibility of reverse causation nor prove the exact cause-and-effect relationships from a cross-sectional survey design. Hence, further study could be extended by collecting longitudinal data through multiple rounds of experiments. Furthermore, several previous studies measured risk perception from a multi-dimensional perspective. Therefore, it would be meaningful to present risk perception as a multi-dimension construct, developing a multi-item scale to promote reliability and validity.

Moreover, this study takes into consideration risk perception that creates social distancing for the adoption of risk management. Some other factors, like knowledge and beliefs of the COVID-19 pandemic, mask-wearing, self–awareness in prevention of COVID-19, number of confirmed COVID-19 cases in a given region, death rate in a given region, and percentage of elderly population in a given region, can also be included in further research. Finally, we considered the mediating and moderating effects of perceived understanding and safety climate. As contingent factors, these effects may interact with other factors, shifting the results conducted in the present study. Besides, several control variables that are associated with population demographics, such as gender, age, and education level, did not show a significant impact on the relationships among these latent variables. This subject, however, is worth exploring in further research.

## 6. Conclusions

This study investigated the impact of risk perception on social distancing during the COVID-19 pandemic. Based on the data collected from an online survey among 317 participants in China throughout May 2020, our analyses indicate that positive changes in social distancing behaviors are associated with increased risk perception, perceived understanding, and safety climate. The individual’s perceived understanding partly plays a positive mediating role in the relationship between risk perception and social distancing behaviors. Furthermore, the safety climate plays a negative role in the relationship between risk perception and social distancing because the safety climate seems to mitigate the effects of risk perception on social distance. Hence, effective health promotion strategies directed at developing or increasing positive risk perception, perceived understanding, and safety climate should be conducted to encourage people to comply with the social distancing policies amid these unprecedented times. Finally, these results are expected to contribute to management guidelines at the level of individual perception and public opinions as well as to assist with effective implementation of the social distancing policies in countries with a high risk of the COVID-19 pandemic.

## Figures and Tables

**Table 1:** Social distancing definitions and rules by country and affiliation.

Country	Affiliation	Social Distancing Definition and Rules
Australia	Government Department of Health	“Physical distancing in public means people should keep 1.5 m away from others wherever possible.” [9]
Brazil	Ministry of Health	“Keep at least 2 m away from anyone who coughs or sneezes.” [10]
Canada	Public Health Agency	“Physical distancing is one of the most effective ways to help prevent the spread of COVID-19, and keep at least 2 arms lengths (approximately 2 m) apart when around other people.” [11]
China	National Health Commission	“Reduce mass gatherings such as activities of entertainment, catering, etc. and stay more than 1 m from others.” [12]
Japan	Ministry of Health, Labour and Welfare	“Carefully avoid 3Cs (closed spaces, crowded places, and close-contact settings) and maintain a distance of at least 1.8 m between people.” [13]
South Africa	National Department of Health	Social distancing refers to limiting public gatherings as much as possible (keep distance at least 1 m). [14]
U.K.	National Health Service	“Avoid close contact with anyone you do not live with at least 2 m (3 steps) away.” [15]
U.S.	Centers for Disease Control and Prevention	“Remaining out of congregate settings, avoiding mass gatherings, and maintaining distance (approximately 6 feet or 2 m) from others when possible.” [16]
	World Health Organization (WHO)	“Maintain at least 1 m (3 feet) distance between yourself and others.” [17]

**Table 2:** Overview of questionnaire items.

Variable	Codes of Measurement Items	Survey Instrument Statements	References
Risk Perception	RP1	My health is at risk during the COVID-19 pandemic.	Dionne et al. [45]; Kim et al. [53]
	RP2	The COVID-19 pandemic is difficult to control.	
	RP3	The coronavirus can cause serious harm to my body once infected.	
	RP4	I think the situation of the COVID-19 pandemic is more serious than previous ones.	
	RP5	I am interested in the pandemic policies implemented by the government. *	
	RP6	I trust that the government recommends the appropriate measures to control the COVID-19 outbreak. *	
	RP7	I am interested in the pandemic information released to the public.*	
Perceived Understanding	PU1	I believe the COVID-19 is caused by the coronavirus.	Qazi et al. [46]; IDSHL [54]
	PU2	I know how people get infected with COVID-19.	
	PU3	I think this coronavirus is a new disease.	
	PU4	I know fever and cough are symptoms of COVID-19.	
Social Distancing	SD1	Avoid going out for any activity due to COVID-19.	Swami V, Barron D [57]; Gudi et al. [58]
	SD2	Avoid contact with individuals who have influenza.	
	SD3	Avoid traveling within or between cities/local regions.	
	SD4	Avoid using public transport due to COVID-19.	
	SD5	Avoid going to crowded places due to COVID-19. *	
Safety Climate	SC1	The government is concerned about the health of people.	Koetke et al. [51]; Neal et al. [55]; Wu et al. [56]
	SC2	I trust the COVID-19 information provided by the government.	
	SC3	There is a clearly stated set of goals or objectives for COVID-19 prevention.	
	SC4	People consciously follow the pandemic prevention regulations.	
	SC5	Being able to provide necessary personal protective equipment for workers during the pandemic.	
	SC6	Offering to workers as much safety instruction and training as needed during the pandemic.	

Note: * Items removed from the initial questionnaire.

**Table 3:** Means, standard deviation, and correlations.

Variable	No. of Items	Mean	S.D.	RP	PU	SD	SC
RP	4	3.68	0.97	-			
PU	4	4.61	0.66	0.32 **	-		
SD	4	4.55	0.69	0.37 **	0.54 **	-	
SC	6	4.45	0.63	0.37 **	0.51 **	0.63 **	-

**Note**: ** *p* < 0.01.

**Table 4:** Results of reliability and validity analysis.

Variable	Code	Factors				Cronbach’s α	CR	AVE
		1	2	3	4			
Risk Perception	RP1				0.61	0.72	0.76	0.47
	RP2				0.53			
	RP3				0.84			
	RP4				0.83			
Perceived Understanding	PU1		0.68			0.856	0.87	0.63
	PU2		0.80					
	PU3		0.78					
	PU4		0.85					
Social Distancing	SD1			0.67		0.814	0.84	0.56
	SD2			0.61				
	SD3			0.76				
	SD4			0.74				
Safety Climate	SC1	0.76				0.881	0.88	0.54
	SC2	0.63						
	SC3	0.76						
	SC4	0.67						
	SC5	0.81						
	SC6	0.73						
Eigenvalue		3.69	3.50	2.44	2.32			
Proportion of Variance (%)		20.51	19.46	13.56	12.88			
Cumulative of Variance (%)		20.51	39.97	53.53	66.41			

**Notes**: Kaiser-Meyer-Olkin (KMO) of sampling adequacy (overall) equals 0.888. Bartlett test (χ^2^ = 3135.94, *df* = 153, *p* < 0.001). Fixing four factors, the extraction method chose Principal Component Analysis by using the correlation matrix. The rotation method chose maximum variance with converging in 25 iterations.

**Table 5:** Goodness-of-fit indices summary of the SEM.

Fit Index	Recommended Value	Estimate
χ^2^/*df*	<3.00	2.912
GFI	>0.90	0.936
AGFI	>0.90 (good) >0.80 (reasonable)	0.896
RMR	<0.05 (good) <0.1 (reasonable)	0.070
RMSEA	≤0.05 (good) <0.08 (reasonable)	0.078
CFI	>0.90	0.949
NFI	>0.90	0.925
TLI	>0.90	0.930
PNFI	>0.50	0.673
PGFI	>0.50	0.576

**Table 6:** The path coefficient values of the SEM.

Dimensions	Unstandardized Path Coefficients	Standardized Path Coefficients	S.E.	C.R.	*p*
RP--->PU	0.150	0.296	0.034	4.435	***
PU--->SD	0.664	0.581	0.079	8.426	***
RP--->SD	0.138	0.238	0.031	4.421	***

**Note**: *** *p* < 0.001.

**Table 7:** Mediating effect (Bootstrapping 5000 times).

Path	Effects	Point Estimation	Product of Coefficients		Bootstrapping			
					Bia-Corrected 95%		Percentile 95%	
			SE	Z	Lower	Upper	Lower	Upper
RP--->SD	Total	0.238	0.052	4.577 *	0.153	0.357	0.146	0.347
	Indirect	0.100	0.040	2.500 *	0.041	0.205	0.036	0.193
	Direct	0.138	0.040	3.450 *	0.072	0.230	0.064	0.219

**Note**: * presents a significant path effect (Z ≥ 1.96).

**Table 8:** Moderated regression analyses of SC.

Model	Variables	Standardized Coefficients	*R^2^*	Change Statistics		Collinearity Statistics	
				Δ*R^2^*	Δ*F*	Tolerance	VIF
1	RP	0.165 ***	0.417	0.42 ***	112.10	0.864	1.158
	SC	0.566 ***				0.864	1.158
2	RP	0.195 ***	0.467	0.05 ***	29.65	0.851	1.176
	SC	0.469 ***				0.745	1.342
	RP × SC	−0.242 ***				0.862	1.160

**Note**: *** *p* < 0.001. VIF represents variance inflation factor (VIF = 1/Tolerance), VIF < 5 (acceptable).

## Supplementary Materials

The following are available online at https://www.mdpi.com/1660-4601/17/17/6256/s1.
